# P140: Interventions aiming to raise healthcare workers adherence to hand hygiene: integrative review

**DOI:** 10.1186/2047-2994-2-S1-P140

**Published:** 2013-06-20

**Authors:** AO Paula, ACD Oliveira

**Affiliations:** 1 UFMG, Belo Horizonte, Brazil; 2 ENB, UFMG, Belo Horizonte, Brazil

## Introduction

Hand hygiene is the most important measure to prevent healthcare associated infections. Although, compliance rates of healthcare workers to such act remain low, making the promotion of hand hygiene through several campaigns necessary.

## Objectives

identify the most frequent interventions used to improve compliance to hand hygiene among healthcare workers.

## Methods

Integrative review of studies published in English, Spanish and Portuguese, indexed in the following databases: LILACS, MEDLINE®, SciELO, Science Direct, Isi Web of Knowlegde and SCOPUS, between the years of 2002 and 2011. The guiding question was: *What interventions have been used to improve compliance to hand hygiene among healthcare workers and what are the results?* The inclusion criteria were: been original and testing any intervention to improve compliance to hand hygiene among healthcare workers. 29 studies were included. An electronic instrument was elaborated in Microsoft Office Excel and descriptive analysis was performed.

## Results

Selected studies presented several methodological discrepancies. The majority of the studies (89.7%) were before-after interventions. Different methods were used to monitor compliance rates (direct observation, product usage and self-reported); 86.2% of the papers implemented a multimodal strategy and the most frequent ones were education (65.5%), feedback and availability of alcohol solution (51.7% each), posters (34.5%), leaders involvement and awards for employees who stood out (13.8%), written information (10.3%), focus group (6.9%), patient empowerment, elaboration of protocols, slogans and seminars (3.4% each) and others (24.1%). There were difficulties in maintaining high rates of compliance after the intervention period.

## Conclusion

Multimodal interventions seems to show better results, but the major challenge faced was not only to increase rates of adherence to hand hygiene, but, mainly, to keep them elevated after the intervention period.

## Disclosure of interest

None declared

